# Shaping of the Present-Day Deep Biosphere at Chicxulub by the Impact Catastrophe That Ended the Cretaceous

**DOI:** 10.3389/fmicb.2021.668240

**Published:** 2021-06-24

**Authors:** Charles S. Cockell, Bettina Schaefer, Cornelia Wuchter, Marco J. L. Coolen, Kliti Grice, Luzie Schnieders, Joanna V. Morgan, Sean P. S. Gulick, Axel Wittmann, Johanna Lofi, Gail L. Christeson, David A. Kring, Michael T. Whalen, Timothy J. Bralower, Gordon R. Osinski, Philippe Claeys, Pim Kaskes, Sietze J. de Graaff, Thomas Déhais, Steven Goderis, Natali Hernandez Becerra, Sophie Nixon, IODP-ICDP Expedition 364 Scientists

**Affiliations:** ^1^UK Centre for Astrobiology, School of Physics and Astronomy, University of Edinburgh, Edinburgh, United Kingdom; ^2^WA-Organic and Isotope Geochemistry Centre (WA-OIGC), School of Earth and Planetary Sciences, The Institute for Geoscience Research, Curtin University, Bentley, WA, Australia; ^3^MARUM-Center for Marine Environmental Sciences, University of Bremen, Bremen, Germany; ^4^Department of Earth Science and Engineering, Imperial College London, London, United Kingdom; ^5^Institute for Geophysics, Jackson School of Geosciences, University of Texas at Austin, Austin, TX, United States; ^6^Department of Geological Sciences, Jackson School of Geosciences, University of Texas at Austin, Austin, TX, United States; ^7^Center for Planetary Systems Habitability, University of Texas at Austin, Austin, TX, United States; ^8^Arizona State University, Eyring Materials Center, Tempe, AZ, United States; ^9^Géosciences Montpellier, Université de Montpellier, CNRS, Montpellier, France; ^10^Lunar and Planetary Institute, Houston, TX, United States; ^11^Department of Geosciences, University of Alaska Fairbanks, Fairbanks, AK, United States; ^12^Department of Geosciences, Pennsylvania State University, University Park, PA, United States; ^13^Institute for Earth and Space Exploration and Department of Earth Sciences, University of Western Ontario, London, ON, Canada; ^14^Analytical, Environmental and Geo-Chemistry, Vrije Universiteit Brussel, Brussels, Belgium; ^15^Department of Earth and Environmental Sciences, University of Manchester, Manchester, IN, United States

**Keywords:** chicxulub, impact crater, deep biosphere, drilling, craters

## Abstract

We report on the effect of the end-Cretaceous impact event on the present-day deep microbial biosphere at the impact site. IODP-ICDP Expedition 364 drilled into the peak ring of the Chicxulub crater, México, allowing us to investigate the microbial communities within this structure. Increased cell biomass was found in the impact suevite, which was deposited within the first few hours of the Cenozoic, demonstrating that the impact produced a new lithological horizon that caused a long-term improvement in deep subsurface colonization potential. In the biologically impoverished granitic rocks, we observed increased cell abundances at impact-induced geological interfaces, that can be attributed to the nutritionally diverse substrates and/or elevated fluid flow. 16S rRNA gene amplicon sequencing revealed taxonomically distinct microbial communities in each crater lithology. These observations show that the impact caused geological deformation that continues to shape the deep subsurface biosphere at Chicxulub in the present day.

## Introduction

Asteroid and comet impact events are known to be able to cause severe disruption to surface-dwelling organisms and ecosystems (Raup, 1992). One such example is the end-Cretaceous Chicxulub impact, which led to the extinction of non-avian dinosaurs and ∼75% of all species (Schulte et al., 2010; Morgan et al., 2016). Despite the growing understanding of the effects of impacts on life, we have little knowledge of how these events, particularly the geological changes caused by them, influence the abundance and distribution of microbial life in the deep subsurface over time as opposed to the microbial changes caused by drastic environmental changes resulting from impact (Bralower et al., 2020; Schaefer et al., 2020). As the deep microbial biosphere has an important role to play in global biogeochemical cycles, such as the carbon cycle (Amend and Teske, 2004; Colwell and Smith, 2013; Magnabosco et al., 2018), it is of considerable interest to investigate how it has been shaped by catastrophic geological events in the past.

In near-surface environments, asteroid impacts have been shown to increase the porosity and permeability of rocks, enhancing microbial colonization (Cockell et al., 2002, 2005; Pontefract et al., 2014; Osinski et al., 2020a). In contrast, sedimentary rocks which often already contain microbially accessible porosity (Friedmann, 1982), may have their porosity reduced by impact, resulting in a loss of colonization space (Cockell and Osinski, 2007). Although these observations inform us about how shock metamorphism and heating affect different rock types, we lack an understanding of how impacts shape the microbial biosphere at the regional scale and how deep subsurface fracturing of rocks, for example, influences the availability of redox couples and nutrients by changing fluid flow at lithological boundaries and within units.

**FIGURE 1:**
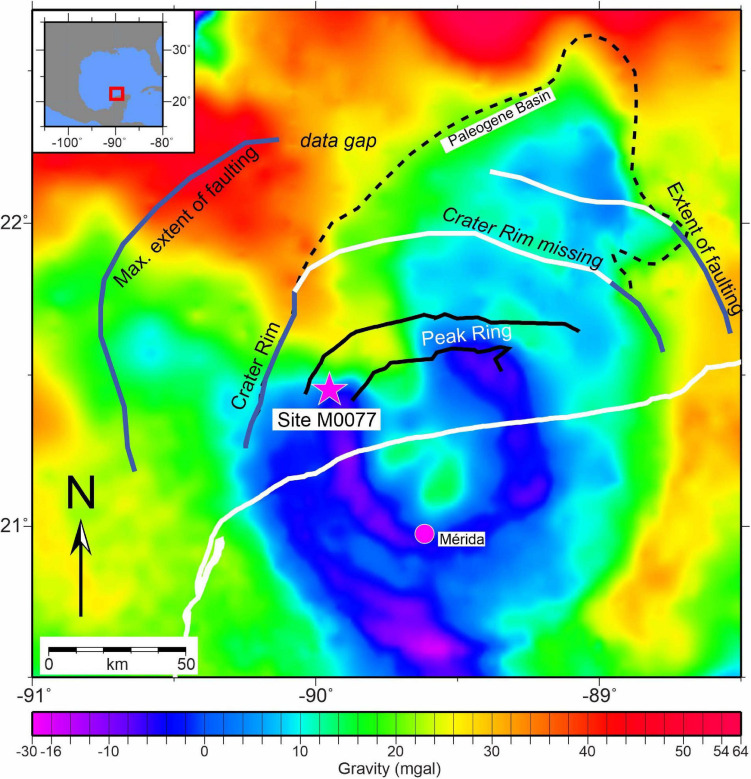
Location of the drilling site M0077 in the Chicxulub crater, Yucatán peninsula, México as seen using gravity data. Figure is adapted from Gulick et al. (2008).

The International Ocean Discovery Program (IODP) and International Continental Scientific Drilling Program (ICDP) Expedition 364 drilled into the Chicxulub crater peak ring, which is a discontinuous topographic ring that is now buried by Cenozoic sediments (Figure 1). The expedition recovered a continuous core (site M0077) from 505.7 to 1,334.7 m below seafloor (mbsf) (Morgan et al., 2016, 2017) and encountered the top of the peak ring at ∼618 mbsf.

In this study, we describe the microbial abundance and community within the Chicxulub crater and discuss how the impact has influenced the distribution of organisms within the present-day structure. We discuss the implications of this work for the role of impacts in planetary processes.

## Materials and Methods

### Core and Sample Collection

Core material was obtained during the IODP and ICDP Expedition 364 (Drilling the Chicxulub K-Pg Impact Crater). Samples were obtained from the major lithostratigraphic units (Gulick et al., 2017; Morgan et al., 2017). Unit 1 consisted of postimpact sedimentary rocks, with subunits defined based on distribution and proportions of different lithologies, as well as specific surfaces indicating erosional truncation of underlying facies or abrupt change in lithology. Units 2 and 3 constitute the upper peak ring material. Unit 2 is suevite with impact melt rock clasts, as well as lithic clasts from the carbonate platform and basement ranging in size from submillimeter to over 25 cm. Unit 3 is composed of impact melt rock with rare (<25%) clasts. As with unit 1, subunits in units 2 and 3 are defined based on distribution of different lithologies and surfaces. Unit 4 is the lower peak ring interval of felsic granitic basement rocks and associated pre-impact subvolcanic rocks as well as suevite and impact melt rock intercalations.

Core sections were retrieved from the core immediately after they had arrived on the drilling rig. The samples were prepared aseptically in the microbiology laboratory prepared for this purpose. To minimize the chance of cross contamination with foreign microbial cells, subsampling was carried out next to a Bunsen burner to create a sterile field. The segments were placed onto baked (8 h, 500°C) sterile aluminum foil, and flame-sterilized with 70% ethanol. The surface was then sprayed with DNA Away (Thermo Scientific) to also degrade contaminant extracellular DNA. Edges were removed using a flame-sterilized chisel, or samples were broken in sterile foil and segments interior to the sample were retrieved. Subsamples of the interior of the core were collected for analysis. Samples of core material (∼1–2 g) were placed into 10 ml sterile vials containing 4 ml sterile 7% NaCl/4% formamide solution for cell enumerations. Samples of core material (∼1–10 g) for cultivation experiments were collected aseptically into 10 ml glass vials and gassed with N_2_ using a sterile cannula. These samples were stored at 4°C until processing. Samples of core material (∼10–50 g) were collected into a sterile Whirl-Pak^TM^ bag, double bagged, and stored at −80°C until return to the laboratory for DNA extraction. These procedures were carried out at least every 9 m through the core. An attempt was made by the geochemistry team to obtain pore water samples for geochemical analysis, but there was insufficient water to obtain samples.

### Core Porosity Determination

Porosity measurements were performed on ∼6 cm^3^ core plugs, approximately once per section (Morgan et al., 2016). Wet and dry samples (dried in a convection oven at ∼105°C for a period of at least 24 h followed by cooling to room temperature in a desiccator for at least 1 h) were weighed using an electronic balance. Volumes of the dried samples were analyzed using a Quantachrome pentapycnometer (helium-displacement pycnometer), provided by MARUM (University of Bremen, Bremen, Germany) (Morgan et al., 2016).

### Borehole Fluid Temperature and *in situ* Temperature Estimates

Borehole fluid temperatures were acquired at the drill site M0077 with a QL40-FTC probe (Morgan et al., 2016) in open borehole conditions (no casing). The tool provides borehole temperature and fluid conductivity measurements from fresh to highly saturated water. Such logs should ideally be acquired after the borehole fluid reached steady state with the ambient environment. During Expedition 364, owing to a tight schedule, the FTC tool was run shortly after the drilling operations and in several phases. The borehole temperatures thus give an underestimate of the *in situ* temperature. The temperature gradient in the top interval (506–700 mbsf) can be used to estimate the *in situ* temperature at depths below (Supplementary Figure 1). Incursions toward lower values (e.g., 936 mbsf) occur locally in the front of fractures, interpreted as opened.

### *In situ* Opened Fracture Determination

Acoustic borehole images were acquired at the drill Site M0077 with a QL40-ABI probe (Morgan et al., 2016). The tool produces millimeter-scale, high-resolution acoustic images of the borehole wall at 360° (Zemanek and Caldwell, 1969; Lofi et al., 2012). Detected fractures are not necessarily connected to deeper or shallower locations since they may be closed or filled away from the borehole wall. Anomalies in the borehole fluid conductivity and temperature logs suggested that at least some of the fractures within the granitoids are a path for active fluid flows in the present day.

### Sulfur Analysis and Bulk X-ray Fluorescence

Homogenized powders of 281 whole rock samples between core sections 3-1 and 303-1 (506.17–1,332.75 mbsf) were measured for total sulfur content by means of a LECO CS-300 carbon sulfur analyzer (Morgan et al., 2016). The same powders were used for bulk energy dispersive X-ray fluorescence (ED-XRF) spectroscopy using a PANanalytical Epsilon 3-XL Benchtop ED-XRF spectrometer. Analytical accuracy and precision of the measurements were assessed by replicate analysis of samples and certified reference granite JG-2 and basalt JB-1b (Morgan et al., 2016).

### Contamination Control

In this drilling expedition, we were not able to add a tracer to the drilling mud. Our means of contamination control was to examine the microbial communities within the drilling mud and seawater mix. Samples of the drilling mud and seawater mix were collected from the drill mud stream in sterile 50 ml Falcon tubes prior to injection into the hole to determine the abundance of microorganisms and the microbial community that would be present on the exterior of cores. Some of this material (∼1–2 g) was preserved as described for core cell enumeration. The remaining material was stored at −80°C for DNA extraction. Samples of drilling mud and seawater mix were acquired every 9 m where possible. In the postimpact material, samples were acquired at 518 mbsf and resumed at 625 mbsf because of complications encountered in drilling in this section.

### Cell Enumerations

Material was collected for cell enumeration as described above. In the case of granitic samples, rocks were gently broken using a sterile mortar and pestle prior to addition to the vials. Suevite and postimpact samples readily disaggregated into a fine powder in the fluid. A volume of two hundred microliters of this material was removed from the vial and diluted if appropriate. An aliquot of a 1 × 1,000 working solution of SYBR^TM^ Gold dye (ThermoFisher Scientific) was added. After incubation for 15 min in the dark, the solutions were filtered using a vacuum pump onto a black sterile 0.22-μm-pore 25-mm diameter polycarbonate filter (Merck Millipore). The filter was transferred to a glass slide, and the slide was viewed under blue light (I3 prism) with a × 100 magnification objective of a fluorescent microscope (Leica DM4000B). Cells were counted in 200 fields of view defined by a 100 × 100 μm grid. The total number of cells was determined and converted into the number of cells per gram of core material, consistent with methods applied in other continental deep subsurface studies (Magnabosco et al., 2018) by conversion based on the mass of core material that was fixed in each sample. The limits of detection for the enumeration were estimated to be 10^4^ cells/g. This was determined from measuring counts using control filters and solutions not containing samples. These background counts were obtained by filtering 200 μl of the NaCl/formamide solution used to preserve the samples for cell enumeration. As with cell enumeration, cells were counted in 200 fields of view defined by a 100 × 100 μm grid. This yielded a background value of 5.2 × 10^3^ (± 2.5 × 10^3^) cells in 200 μl. We note that this empirical value is consistent with theoretical calculations (Kallmayer et al., 2008). Assuming that most samples were an approximately 1:10 sediment slurry, and given the use of 200 μl of slurry, the theoretical detection limit should be ∼5 × 10^3^ cells/cm^3^ or ∼1 × 10^3^ cells in 200 μl.

### Enrichment Cultures

The enrichment cultures were prepared after the return of the samples to the laboratory 30 days after the end of drilling. In the Cockell lab, samples of core material of ∼1.5 g were dispensed into 10 ml serum bottles in a Coy anaerobic chamber (Coy Laboratory Products, United States) and 1 ml of media was added. The amounts of media added were chosen to maximize the rock to fluid ratio, generating a slurry of core material. The main purpose of the media was to provide minor additional nutrients and redox couples. The following media were used:

#### Subsurface Medium I (MM1)

To supply CHNOPS elements and redox couples (iron and sulfate reduction) to enrich for anaerobic respirers and fermenters. NH_4_Cl (3.7 mM), K_2_HPO_4_ (1.1 mM), Na_2_SO_4_ (1.4 mM), and iron citrate (0.41 mM). The following carbon sources, all at 0.2 g/L: yeast extract, peptone, casamino acids, and sodium acetate. Samples were gassed with a 100% N_2_ or 20% CO_2_/80% N_2_ headspace.

#### Subsurface Medium II (MMII)

To supply CHNOPS elements and redox couples (iron, sulfate, and nitrate reduction) to enrich for anaerobic respirers and fermenters. A richer version of MM1. NH_4_Cl, K_2_HPO_4_, and Na_2_SO_4_ the same as MM1, but with KNO_3_ (2.0 mM) and iron citrate (4.0 mM). The following carbon sources, all at 0.2 g/L: yeast extract, peptone, sodium acetate, casamino acid, sodium formate, xylan, fructose, and sodium pyruvate. NaHCO_3_ (1.25 g in 25 ml) was autoclaved separately and added. Samples were gassed with a 20% CO_2_/80% N_2_ headspace.

#### Simple Organics Medium (Org)

To enrich for organisms that might use small organic molecules as an electron donor or to ferment. NH_4_Cl (18.7 mM), K_2_HPO_4_ (5.7 mM), KCl (6.7 mM), and MgSO_4_ × 7 H_2_O (2.2 mM). The following carbon sources, all at 1.0 g/L: sodium gluconate, sodium acetate, sodium formate, and sodium fumarate. Samples were gassed with an 80% CO_2_/20% N_2_ headspace.

#### Subsurface Chemolithotroph Medium (Chemolith)

To enrich for chemolithotrophs. NH_4_Cl (37.4 mM), K_2_HPO_4_ (11.5 mM), KNO_3_ (19.8 mM), Na_2_SO_4_ (14.1 mM), iron citrate (4.1 mM), and NiCl_2_ × 6H_2_O (3 mg/L). NaHCO_3_ (1.25 g in 25 ml) was autoclaved separately and added. Samples were gassed with an 80% H_2_/20% CO_2_ headspace.

#### Organics/H_2_ Medium (OrgH)

To enrich for organisms using H_2_ as the electron donor or organics. NH_4_Cl (18.7 mM), CaCl_2_ (1.0 mM), K_2_HPO_4_ (2.3 mM), MgCl_2_ × 6H_2_O (1 mM), MgSO_4_ × 7H_2_O (0.09 mM), and yeast extract (1.0 g/L). NaHCO_3_ (1.25 g in 25 ml) were autoclaved separately and added. The following were also added as typical constituents of anaerobic media to provide additional nutrients: 10 ml Wolfe’s trace element solution, 10 ml Wolfe’s vitamin solution, and 1 ml tungstate-selenate solution. Samples were gassed with an 80% H_2_/20% CO_2_ headspace.

All enrichment samples were incubated for 8 months. Media without sample material was incubated to confirm that media was not contaminated. Core material shallower than a depth of 802 mbsf (core 114) were incubated at 50°C, samples between 802 and 1124 mbsf (core 235) were incubated at 60°C, and samples between 1,124 mbsf to the bottom of the core (depth of 1,333 mbsf, core 303) were incubated at 70°C. Incubation was performed at temperatures expected to be close to *in situ* conditions. These were derived from downhole logging measurements of the *in situ* borehole fluid temperature (see “**Borehole fluid temperature and *in situ* temperature estimates**”). A total of 240 enrichments were established. At 50°C, 23 × MM1, 42 × MM2, 21 × Chemolith, 21 × OrgH, 10 × Org; at 60°C, 37 × MM2, 8 × Chemolith, 8 × OrgH, 9 × Org; at 70°C, 12 × MM1, 23 × MM2, 8 × Chemolith, 8 × OrgH, 10 × Org.

After incubation, samples were vigorously vortexed and 50 μl of fluid was removed under anaerobic conditions. Five microliters of a 1 × 1,000 working solution of SYBR^TM^ Green DNA binding dye (Invitrogen, United Kingdom) was added, and the sample was left for 15 min. Samples were then analyzed for the presence of cells by fluorescence microscopy.

Samples that were found positive for cells were then DNA extracted using the DNeasy PowerSoil Kit (QIAGEN, Germantown, MD, United States). 16S rRNA gene libraries were prepared for subsequent MiSeq Illumina sequencing using the primers 28F (GAGTTTGATCNTGGCTCAG) and 388R (TGCTGCCTCCCGTAGGAGT) targeting the V1–V2 region. Samples were sequenced by RTL Genomics (Lubbock, Texas, United States). Bioinformatic analysis was undertaken using QIIME2 as described in detail below. We sequenced 10 of the enrichments that did not support any microscopically observed growth and one sample of each of the media used for enrichment. DNA sequences observed corresponded to those expected from kit contaminants (e.g., *Streptococcaceae*, *Staphylococcaceae*) (Salter et al., 2014; Sheik et al., 2018) but no other microorganisms.

The media in which reported enrichments were achieved were as follows: 50°C enrichments: *Aeromonas* (MM2) and *Acidiphilium* (MM1); 60°C enrichments: *Desulfovermiculus* (MM2), *Desulfovermiculus* (Org), *Enhydrobacter* (MM1), and *Brockia* (OrgH). No enrichments yielded organisms at 70°C.

### DNA Extraction From Core and Drilling Mud Material

In the Coolen lab at Curtin University, DNA extraction was carried out to obtain the total environmental DNA from the whole sample, irrespective of whether it was from inactive or active organisms. For the analysis of core material, the following extraction procedure was used: core material (∼10 g pieces from the center of the core) was defrosted aseptically in a HEPA-filtered horizontal laminar flow hood (SafeGuardHLF^TM^). Prior to DNA extraction, possible remaining traces of contaminant surficial DNA on the rock samples, which were already aseptically sampled offshore, was crosslinked for 10 min on each side inside a UVLink Ultraviolet crosslinker (UVITEC Cambridge) by placing the pieces of rock 1 cm away from the 5 × 8 W 254 nm bulbs. The UV-sterilized pieces of rock (∼5–10 g) were then pulverized using a heat-sterilized (8 h at 500°C) mortar and pestle. This material was then used for DNA extraction using the DNeasy PowerMax Soil Kit (QIAGEN) with modifications after Direito et al. (2012) to ensure efficient release of mineral-adsorbed DNA. As part of the modified protocol, the kit’s Powerbead Solution was replaced by 1 M Na_2_HPO_4_ (pH 9.5, not adjusted) and 15 vol% molecular-grade ethanol. Prior to adding ethanol to the modified bead solution, possible traces of contaminant DNA were removed from the 1 M phosphate buffer through centrifugation over a 30-kDa Amicon Ultra-15 centrifugal system unit (MilliporeSigma, Billerica, MA, United States). After adding the kit’s buffer C1, the bead tubes containing the modified bead solution and pulverized rocks underwent an additional freeze–thaw lysis step (15 min at 65°C followed by freezing at −80°C and thawing under rotation at 50°C). The thawed samples were homogenized for 60 s at 1,600 rpm using a 50-ml tube adapter inside a FastPrep 96 homogenizer (MP Biomedicals, Irvine, CA, United States). Subsequent extraction followed the standard protocol of the DNeasy PowerMax Soil Kit. The extracted DNA was concentrated using Amicon^®^ Ultra-15 Centrifugal Filter Devices (30 kDa) and purified with OneStep^®^ PCR Inhibitor Removal Kit (Zymo Research, Irvine, CA, United States). Five extractions without sample [extraction controls (EC)] served as controls for the presence of contaminants in the kit’s reagents and contaminants that were introduced during the processing of the samples. The same extraction procedures were used to extract DNA from 5 ml of the drilling mud (DM) samples and concentrated seawater used during coring. The concentration and quality of the extracted DNA was determined by spectrometric measurements using a NanoDrop^TM^ 3300 Spectrofluorometer (ThermoFisher Scientific, Waltham, MA. United States) and agarose gel electrophoretic analysis.

### 16S rRNA Gene Library Preparation

For the preparation of quantitative PCR mixtures, aliquots of extracted and purified DNA from the various rock samples and controls were added to 20 μl reaction mixtures with 1 × Green^TM^ Premix Ex Taq^TM^ (Tli RNaseH Plus) (Takara Bio Inc) and 0.2 μM final concentration of primers U519fM (Wuchter et al., 2013) and U806R (Caporaso et al., 2012) targeting the V4 region of bacterial and archaeal environmental 16S rRNA genes (Caporaso et al., 2012). The amount of SYBR^TM^ Green-stained double-stranded amplicons was followed real time using a Realplex quantitative PCR cycler (Eppendorf). The forward and reverse primers included the Illumina flowcell adapter sequences as well as the pad regions. The reverse primer contained a unique 12 base Golay barcode sequence to support pooling of samples (Caporaso et al., 2012). Cycling conditions included the following: initial melting (95°C for 60 s) and 25–35 cycles consisting of a melting step (95°C for 5 s), annealing (60°C for 30 s), and primer extension plus imaging (72°C for 60 s). The reactions were stopped at the end of the exponential phase to minimize over-amplification and the formation of artefacts. The amount of DNA in each barcoded amplicon was measured fluorospectrometrically (Picogreen assay) using a VersaFluor^TM^ Fluorometer (BIO-RAD Laboratories), and equimolar amounts were pooled. The pooled library was then concentrated using an Amicon Ultra 0.5 30 kDa Centrifugal Filter (MilliporeSigma) and subjected to agarose gel electrophoresis. After imaging of the SYBR^TM^ Green-stained gel using a blue light transilluminator (Clare Chemical, Dolores, CO, United States), the desired amplicon was gel-purified using the Monarch^®^ DNA Gel Extraction Kit (New England Biolabs) and sent to the Australian Genomic Research Facility (AGRF) for subsequent paired end (2 × 300 bp) Illumina MiSeq sequencing.

### Bioinformatics and Biostatistics

The obtained IIlumina MiSeq reads were processed using the Quantitative Insights into Microbial Ecology 2 (QIIME2, version 2020.11^1^) pipeline (Bolyen et al., 2019). The raw paired-end reads were demultiplexed using q2-demux. Primer and Illumina adapter sequences were removed using q2-cutadapt (Martin, 2011), followed by denoising and chimera removal using the Divisive Amplicon Denoising Algorithm (DADA2) plugin (Callahan et al., 2016). QIIME2 feature-classifier classify-sklearn (Pedregosa et al., 2011) was used for the taxonomic annotation of the high-quality Amplicon Sequence Variants (ASVs) against the SILVA 138 database (Silva-138-99-515-806-nb-classifier.qza) (Supplementary Table 2; Quast, 2013).

Contaminant ASVs that were present in the rock samples as well as in the various controls (drilling muds, sea water, and procedural blanks) were removed from the ASV × sample abundance matrix and analyzed separately (Supplementary Table 6). This included the stringent removal of rare ASVs that could have represented undetected contaminants in the controls. Therefore, only ASVs that occurred more than 10 times in at least one sample were considered for downstream analysis.

A Venn diagram prepared in the online program Gene List Venn Diagram^2^ was used to reveal the number of microbial taxa assigned at the lowest identified taxonomic levels that were unique to or shared between the three lithologies. Canonical analysis of principal coordinates (CAP) was performed in the biostatistical package PRIMER E vs. 7 (Clarke and Gorley, 2015) to show the spatial distribution of microbial communities at the lowest assigned taxonomic level in the three lithologies. This analysis was performed using Bray-Curtis similarity of standardized and square root transformed data. Vector overlays were drawn to show the dominant significant indicator species (> 5% of total indicator taxa) for each lithology. Pairwise permutational multivariate analysis of variance (PERMANOVA) and the Monte-Carlo permutation procedure with 999 permutations in PRIMER E vs. 7 was used to reveal whether the communities differed significantly between the lithologies. Indicator species analysis was performed using the R package IndicSpecies (DeCáceres and Legendre, 2009). Indicator values were calculated with normalized and square root transformed ASV data using the multipatt function, with duleg = true, 999 permutations, and alpha value = 0.05. The resulting indicator species associated with postimpact, suevite, and granitic basement samples were projected as vectors in the CAP plot. Similarity percentage (SIMPER) analysis was performed in PRIMER-e v7 using Euclidean distance of normalized and square root transformed data to study the contributions of the in-parallel-measured environmental and geochemical parameters [total organic carbon (TOC), S, Mn and Fe, porosity, and *in situ* temperature] to the three major lithologies (Supplementary Table 6). The sequence data has been submitted to the sequence read archive (SRA) of the National Center for Biotechnology Information (NCBI) under BioProject number PRJNA726950 entitled “Chicxulub Impact Crater Microbiome.”

## Results

### Microbial Abundance in Suevites

**FIGURE 2:**
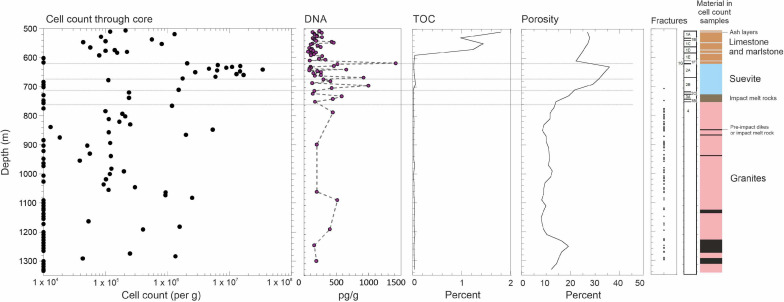
Microbial abundance through the Chicxulub Expedition 364 (site 0077A) core. Diagram showing cell enumerations alongside total extracted DNA and total organic carbon (TOC) content, porosity, and open fractures. The limit of detection is taken as 1 × 10^4^ cells/g. The horizontal lines separate the sections of suevite. TOC and porosity are displayed as moving averages of each 20 m depth. The classification of the geological units adopted by the drilling expedition scientists are shown alongside (see section “Materials and methods”).

Cell enumerations were begun at 506 mbsf in the postimpact sequence and continued at 9 m intervals throughout the core, although in some locations additional samples were studied (Supplementary Table 1). Above the crater, in the Paleogene postimpact sedimentary sequence (lithostratigraphic units 1E to 1G; Figure 2; Morgan et al., 2017), cell enumerations are highly variable with depth to 617.34 mbsf with a maximum abundance measured at 518.67 mbsf of 1.3 × 10^6^ cells/g wet weight. Through the sequence, there is a downhole increase in rock density (from 2.0 to 2.5 g/cm^3^) and reduction in porosity (25–35% to 10–15%) (Christeson et al., 2018) of the rocks (Figure 2), which weakly correlates with cell abundance (*r* = 0.47, *p* = 0.05). TOC values are variable in this interval (mean, 1.03%; SD, ± 0.90; Figure 2) and are not correlated with cell abundance (*r* = −0.27, *p* = 0.278).

Transitioning into the impactite lithologies (suevite Unit 2A starting at ∼617.33 mbsf, Figure 2), we observe a marked increase in cell abundances at the top of the suevite, which exceed 1 × 10^7^ cells/g wet weight (see “**Materials and Methods**” for unit descriptions). This sorted suevite was formed by ground surge, explosive interactions between hot impact melt and seawater (Osinski et al., 2020b), and lateral transport of material into the crater, and settling within a flooded impact basin immediately following impact. The suevite contains clasts of impact melt rock, sedimentary rock, and basement lithologies, embedded in a fine-grained matrix, predominantly of clay and calcite. Contamination of our cell counts is unlikely because we observe cell counts in some of our samples that exceed those from the drilling mud (Supplementary Figure 2).

Cell numbers decline at the bottom of unit 2A near a suggested erosional contact within the suevite (Gulick et al., 2019). Both cell numbers and porosities are generally lower in suevite subunits 2B and 2C compared with the overlying subunit (2A). The porosity further reduces to ∼20% at the bottom of subunit 2C at depth 721.61 mbsf (Figure 2), which has been reinterpreted to be a breccia derived from, or transitional to, clast-rich impact melt rock (Figure 2; Gulick et al., 2019). Throughout the suevite, decreasing cell abundances correlate to decreasing porosity (*r* = 0.49, *p* = 0.008).

We observe increases in cell numbers at unit interfaces, such as between suevite units 2B and 2C (cell numbers 1.5 × 10^6^/g) where increasing clast size is noted and between impact melt rock unit 3B and impact-uplifted granitic basement unit 4 (cell numbers 1.2 × 10^6^/g).

### Microbial Abundance in Impact-Shocked Granites

**FIGURE 3:**
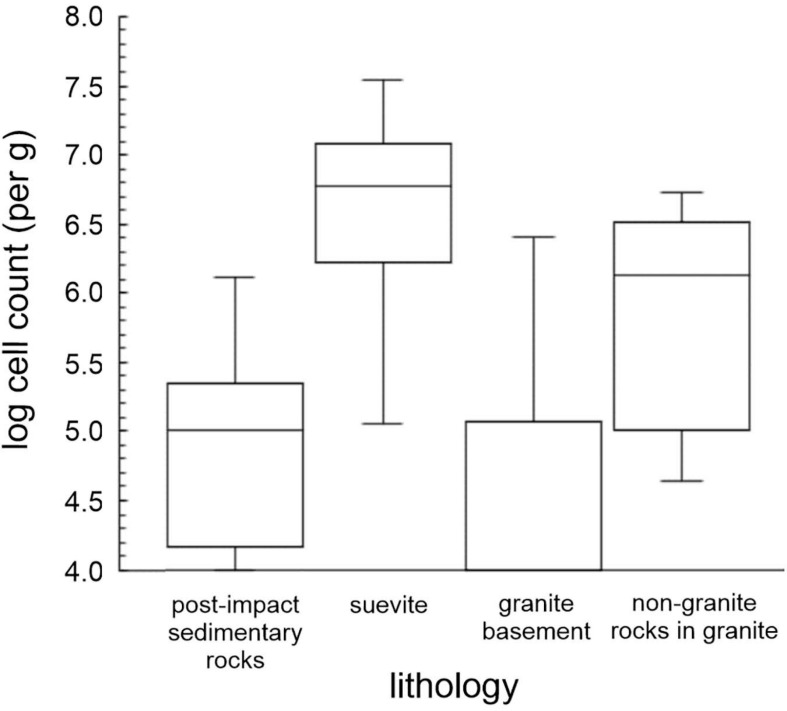
Box plots of cell numbers associated with Chicxulub impact lithologies. Lithologies shown are postimpact sedimentary rocks, suevite (cell enumerations in all suevite units), granite basement alone (elevated cell counts in non-granitic material removed), and non-granite rocks in the granitic unit (i.e., geological interfaces within the granitic unit). Logarithmically transformed data are shown as a box plot.

Below ∼766 mbsf in the granitic basement material (unit 4), we find low or undetectable cell abundances (Figures 2, 3) in most of the samples analyzed. Our data are the first microbiological analysis of a deep continuous core from continental granites. Previous studies of deep subsurface granites have been localized to point sampling in locations such as boreholes and deep mines where there is a well-established water flow. In these environments, cell numbers are typically on the order of ∼10^4^ cell/ml (Pedersen and Ekendahl, 1992; Pedersen, 1997; Fukuda et al., 2010; Purkamo et al., 2013) consistent with the low abundances that we observe.

Nevertheless, we observed regions in the granites with elevated cell abundances (Figures 2, 3). These locations correspond to non-granitic rocks such as strongly serpentinized preimpact subvolcanic, ultramafic basanite/dolerite (847.57 and 865.20 mbsf), and places where suevite and impact melt rock were intercalated into the granites during crater formation, containing a range of mafic gneiss lithologies (1,274.20–1, 291.17 mbsf).

### Microbial Enrichment Experiments

Cultivation experiments using media targeting different groups of organisms including heterotrophs (anaerobic respiration and fermentation) and chemolithotrophs (see “**Materials and methods**”) resulted in 240 enrichments. Of the 240 enrichments, six yielded growth of organisms consistent with the challenge of enriching organisms from oligotrophic deep subsurface environmental samples (Amman et al., 1995; Pedersen, 1997). Two of the enrichments grew at 50°C. One of these enriched an organism affiliated with *Acidiphilium* (Alphaproteobacteria, Acetobacterales) (631.47 mbsf; suevite) and the other affiliated with *Aeromonas* (Gammaproteobacteria; Aeromonadales) (649.79 mbsf depth; suevite). Both organisms were enriched in heterotroph media suggesting anaerobic respiration or fermentative metabolisms. Four enrichments grew at 60°C. One organism was affiliated *Desulfovermiculus* (Desulfovibrionia; Desulfovibrionales) (701.03 mbsf; suevite and 829.29 mbsf; granite), which performs sulfate reduction, consistent with our observation of a black sulfide precipitate in the cultures. Sulfides are observed in the core at these depths at ∼0.1 wt% abundance (Supplementary Figure 1). One was affiliated with *Enhydrobacter* (Gammaproteobacteria, Pseudomonadales) (829.29 mbsf; granite), which performs heterotrophic or fermentative metabolism (Staley et al., 1987), and *Brockia* (Thermoanaerobacteria, Thermoanaerobacterales) (847.57 mbsf; basanite/dolerite), which performs thermophilic iron reduction or fermentation (Zeikus et al., 1979). No enrichments yielded organisms at 70°C. TOC concentrations are low in the suevite and granites (typically less than 0.1%; Figure 2) but may provide the primary carbon and energy source for these organisms.

### Microbial Community Analysis

**FIGURE 4:**
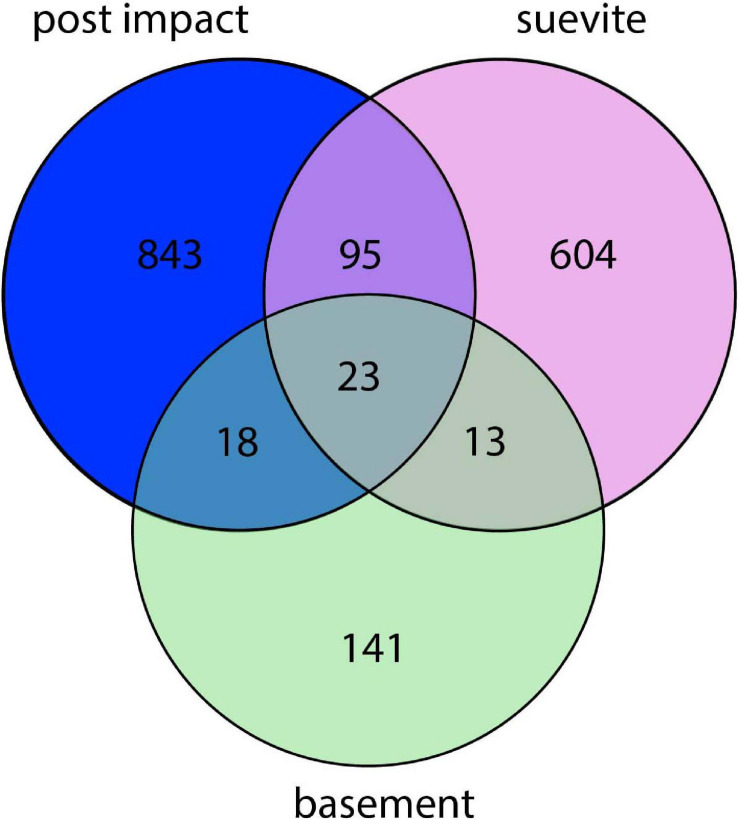
Venn diagram showing the number of microbial taxa (assigned at the lowest identified taxonomic levels) in the three lithologies. The non-overlapping parts of the circles show the number of taxa that are unique to each lithology, whereas the overlapping parts of the circles display the number of taxa that are shared between the lithologies.

In total, 3,090 ASVs were recovered from all samples combined and 2,757 more abundant ASVs (∼90%) remained after the stringent removal of rare ASVs which did not occur more than 10 times in at least one of the analyzed samples (Supplementary Table 2). After stringent quality control, 1,737 ASVs were identified as being indigenous to the various analyzed rock samples, and a total of 1,018 contaminant ASVs, which occurred also in the various controls (drilling muds, sea water, and procedural blanks), were removed from the dataset and analyzed separately (Supplementary Figures 3, 4). A total of 843, 604, and 141 ASVs were unique for the postimpact sediments, suevite, and granitic basement intervals, respectively. Figure 4 shows a Venn diagram of the number of ASVs that were shared between these lithologies. Throughout the core, 89 and 38% of the bacterial ASVs could be assigned to respectively genus and species level. Grouping of ASVs that were assigned at the same lowest possible taxonomic level yielded 709 unique taxa in total. The highest number of taxa (136) was observed at a depth of 616.18 mbsf, 85 cm above the transition between the postimpact sediments and suevite at a depth of 617.33 mbsf (Supplementary Figure 5A). The remainder of the postimpact sediments contained between 7 and 77 taxa. Samples from the suevite and granitic basement intervals contained 12–72 and 18–37 taxa, respectively (Supplementary Figure 5A).

**FIGURE 5:**
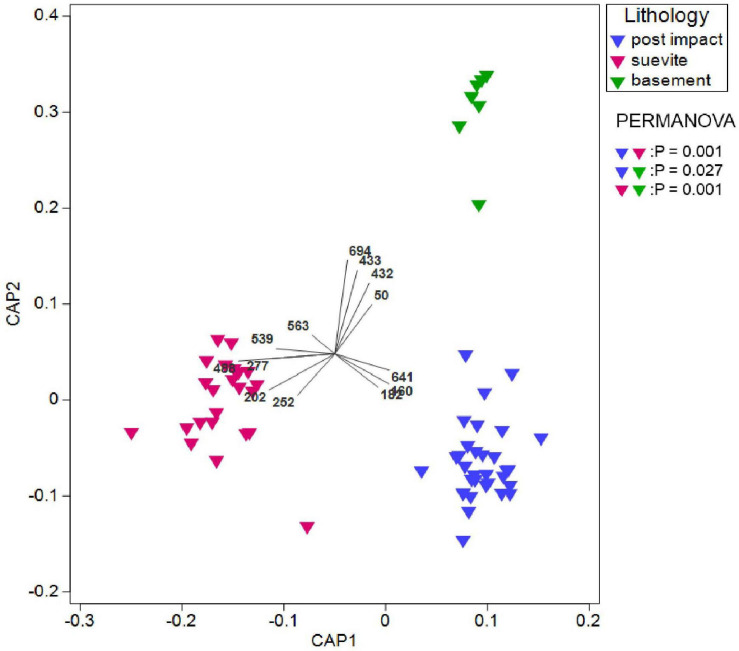
Canonical analysis of principal coordinates (CAP) showing the spatial distribution of the microbial communities at the lowest taxonomic level in the three crater lithologies. Also shown are the probabilities for pairwise comparisons of the crater units. Vector overlay shows the dominant significant indicator species for each lithology. For details about the ISA, see Supplementary Tables 3, 4.

Permutational multivariate analysis of variance (PERMANOVA) revealed statistically significant differences between bacterial communities in postimpact sediments vs. suevite (*P* = 0.001), postimpact sediments vs. granitic basement (*P* = 0.027), as well as between the suevite and granitic basement (*P* = 0.001) (Figure 5 and Supplementary Table 3).

**FIGURE 6:**
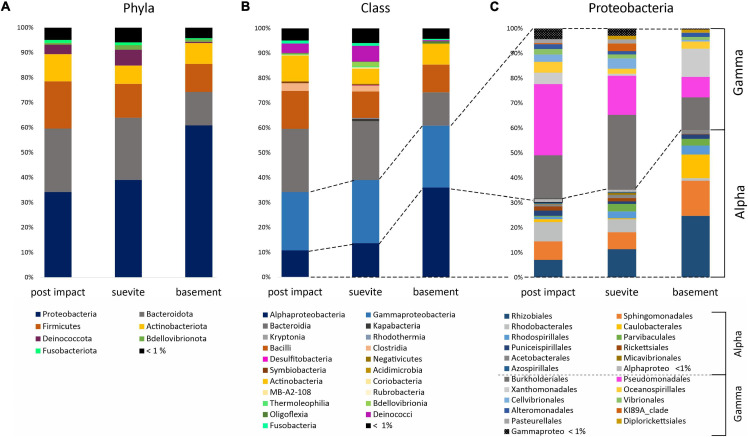
Bar graphs showing the average distribution of the major phyla **(A)**, classes **(B)**, and orders for Proteobacteria **(C)** in the three lithologies: postimpact Cenozoic interval (*n* = 32 samples), suevite (*n* = 23), and granitic basement (*n* = 7). See Supplementary Figure 5 for details on the relative abundance of the main phyla and classes in the three lithologies.

Proteobacteria, Bacteroidota, Firmicutes, Actinobacteria, Deinococcota, and Bdellovibrionota comprised ∼95% of the dominant phyla in the three lithology categories (Figure 6A and Supplementary Figure 6). Proteobacteria was the most dominant phylum in all three major lithologies. In the granitic basement interval, Proteobacteria were more abundant than in the postimpact sediments and suevite (Figure 6A and Supplementary Figure 6). At class level, Gammaproteobacteria dominated over Alphaproteobateria in postimpact and suevite intervals, whereas this distribution was reversed in the granitic basement (Figures 6B,C and Supplementary Figure 6). Bacteroidota was the second most abundant phylum and was dominated by the class Bacteroidia especially in the postimpact and suevite samples (Figures 6A,B and Supplementary Figure 6). Kapabacteria (formerly known as non-photosynthetic Chlorobi-related uncultured OPB56 clade), Kryptonia, and Rhodothermia comprised a small fraction of Bacteroidota and were almost exclusively present in the suevite interval (Figure 6B and Supplementary Figure 6). Firmicutes were most abundant in the postimpact interval (Figure 6A). At class level, Bacilli comprised 99.5% in the granites and ∼80% in the postimpact and suevite samples. Clostridia represented the second most abundant class within the Firmicutes and was mainly present in the postimpact and suevite samples (Figure 6B and Supplementary Figure 6). Only a small percentage of the classes Symbiobacteria and Negativicutes were present in the postimpact and suevite intervals and Desulfobacteria were only present in the suevite samples (Figure 6B and Supplementary Figure 6). The phylum Actinobacteriota was roughly equally distributed in the three lithologies (Figure 6A). Actinobacteria comprised 95 and 80% of Actinobacteroita in the postimpact and suevite intervals, respectively, and was the only class within this phylum that could be identified in low abundance from the basement samples (Figure 6B and Supplementary Figure 6). A small contribution of the classes Thermoleophila, Rubrobacteria, Acidimicrobiia, MB-A2-108, and Coriobacteria (Actinobacteriota) were detected only in the postimpact sedimentary rocks and suevite samples (Figure 6B and Supplementary Figure 6). Lastly, the phylum Deinococcota was mainly present in the postimpact rocks and suevite intervals (Figure 6A).

### Indicator Species Analyses

Indicator species analyses (ISA) based on a comparison of the distribution of ASVs that were grouped at the lowest taxonomic level in all three lithologies (postimpact, suevite, and granitic basement) did not render statistically significant indicator species for the postimpact section. However, pairwise ISA (postimpact sediments vs. suevite) revealed unclassified Flavobacteriaceae (Bacterioidota; ISA #160 and 182) and *Halomonas* (Gammaproteobacteria; Halomonaceae; #641) as significant indicator taxa for the postimpact interval (Figure 5 and Supplementary Tables 4, 5). Both groups comprised 90 and 10%, respectively of the total reads from indicator species (Supplementary Tables 4, 5).

ISA revealed five indicator species for the suevite interval, which belong to the phyla/classes Bacteroidota, Firmicutes, Deinoccota, and Alpha- plus Gammaproteobacteria (Figure 5 and Supplementary Table 4). Indicator species were from the families (ID No; percentage of taxa) env.OPS_17 (#202; ∼56%), Thermaceae (#252; ∼24%), Bacilliaceae (#277; ∼6%), Stappiaceae (#488; ∼11%), and Kapabacteriales (#208; ∼4%) (Figure 5 and Supplementary Table 4). In the granitic basement section, 14 indicator species were identified and composed of the phyla/class Actinobacteriota, Bacteroidota, Alpha- and Gammaproteobacteria, and from the domain of the Archaea (Bathyarchaeia). Dominant indicator species (> 5% from the total indicator species) were derived from the families Caulobacteriaceae (#432 and 433; ∼31%), Comamonadaceae (#563; ∼26%), Geothermatophilaceae (#50; ∼8%), Sphingomonadaceae (#539; ∼7%), and Xanthomonadaceae (#694; ∼6%) (Figure 5 and Supplementary Table 4).

### Environmental Parameters

**FIGURE 7:**
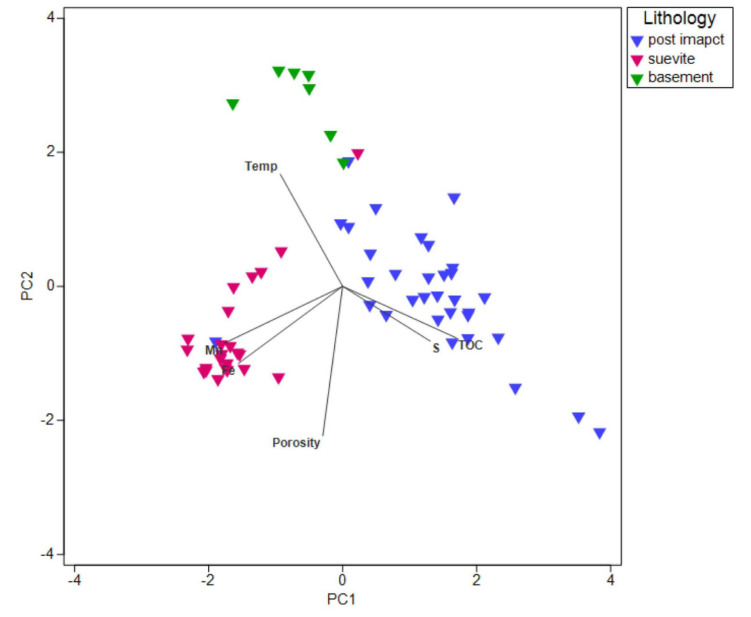
PCA plot based on SIMPER analysis of environmental parameters (porosity, TOC, temperature, Fe, S, and Mn). Vectors overlay represents each of these parameters contributing to each lithological interval.

Principal component analysis (PCA) and SIMPER analysis were used to visualize and investigate the contribution of environmental parameters (Figure 2 and Supplementary Figure 1) in each lithological interval (Figure 7 and Supplementary Table 6). Analyzed environmental parameters were porosity, TOC, temperature, Fe, and S (biologically essential elements) and Mn (biologically essential trace elements). In the postimpact sedimentary rocks, S (38.4%) was the most prominent environmental contributor followed by TOC (29.2%), porosity (15.8%), and Mn (13.5%). Porosity (53.7%) appeared to be the most dominant environmental contributor in the suevite followed by Mn (27.8%) and Fe (15.6%). In the basement granite, temperature (84.2%) was the main environmental contributor followed by porosity (7.7%) and Mn (4.3%) (Figure 7 and Supplementary Table 6).

## Discussion

### Impact-Induced Enhancement of Cell Abundance

Drilling of the Chicxulub impact crater allowed us to investigate the influence of the impact aftermath on the deep subsurface abundance of microorganisms through the three key lithologies of the crater, namely postimpact sedimentary rocks, the suevites (IUGS, 2007) and the underlying uplifted granitic rocks. In the postimpact sedimentary section, cell numbers were comparable with other deep subsurface sedimentary rock settings where cell abundances from 10^3^ to 10^6^ cells/ml have typically been observed (Shimizu et al., 2006; Kato et al., 2009).

We observed a marked increase in cell abundance at the interface between the postimpact sedimentary rocks and the suevite. An explanation for the increase in cell abundance is the preferential channeling of fluids, and thus potentially nutrients and energy supplies, between the less-permeable limestone/marl layers above the crater and the underlying higher porosity suevite, despite the relatively low TOC in the suevite compared with the postimpact sedimentary rocks. These impact-altered, elevated-porosity materials are thought to have allowed for fluid flow, leading to their hydrothermal alteration after the impact (Simpson et al., 2020). The high porosity of the suevite (∼35%), higher than typical marine sedimentary environments (Parkes et al., 2005; Tanikawa et al., 2018), is directly linked to the deposition of this material by ocean resurge prior to its eventual burial after the impact (Christeson et al., 2018). These data demonstrate that the impact produced new lithological horizons and deposits that caused a long-term improvement in the colonization potential for deep subsurface microorganisms. Microbial abundances have been shown previously to be higher at interfaces between geological units (Parkes et al., 2005) because subsurface environments often restrict nutrient supply (Tanikawa et al., 2018). Our data demonstrate this phenomenon at impact-generated geological interfaces.

### An Impoverished Biota in the Impact-Altered Granites

One factor influencing the low cell abundances observed in the deep subsurface granitic material could be energetic limitation. Deep crustal environments are generally deprived of electron donors for microbial growth. Unlike deep subsurface marine sediments, for example, granites would be expected to have low organic carbon availability. TOC values throughout the granitic section were less than 0.1%. Granitic rocks cannot react like deep subsurface mafic and ultramafic rocks in serpentinization reactions to produce hydrogen, another electron donor for deep life (Sherwood Lollar et al., 2007). Thus, in the absence of exogenous subseafloor fluid flow, granitic rocks would be predicted to be poor substrates for chemolithotrophy.

However, the granites in the Chicxulub core are atypical since they have been shocked and fractured during impact. These geological changes might be expected to enhance fluid flow and microbial access. The material has lower mean densities of 2.39–2.44 g/cm^3^ compared with typical granitic values of 2.62–2.67 g/cm^3^. The mean porosity is 11%, significantly higher than typical values of < 1% (Christeson et al., 2018). Impact-induced fracturing has been shown to facilitate microbial colonization in surface exposed rocks (Cockell et al., 2002, 2005; Pontefract et al., 2014) and in the subsurface (Cockell et al., 2012).

The low cell abundances measured through much of the granitic unit, despite its high impact-induced porosity and fracturing, may be caused by the impact history of the material. During impact, the granites were rapidly transported from mid-crustal depths to the near surface. Numerical modelling of the Chicxulub impact event suggests that these rocks were sourced from 8 to 10 km depth (Vermeesch and Morgan, 2008; Morgan et al., 2016). Assuming a geothermal gradient of ∼20°C in the continental deep subsurface (Colwell and Smith, 2013), these rocks would likely have been at temperatures of ∼160–200°C prior to impact, well above the currently accepted upper-temperature limit for life of 122°C (Takai et al., 2008), and therefore sterile before impact. The release of additional heat during the passage of the impact-generated shock wave would have further exposed the granitic rocks to sterilizing conditions. Hydrothermal minerals in the core suggest postimpact temperatures of ∼355–425°C with temperatures of at least 250°C sustained for at least 200,000 years (Kring et al., 2020). Both the preimpact conditions and impact-induced sterilization of the granites suggest that the rocks have only been amenable to microbial colonization for < 66 myr. The modern biosphere could only have been re-established since that time where fractures and fluid flow have allowed colonization.

### Enhancement of Cell Abundances at Interfaces Within the Granitic Rocks

Despite the low cell abundances observed in the bulk granitic rocks, we observed enhancements in cell abundances not only in non-granitic rocks which included preimpact rocks but also locations where suevite and impact melt rock, containing mafic gneiss lithologies, were intercalated into the granites during crater modification. This observation is a further instance of enhanced colonization linked to the geological effects of the impact. The preferential colonization of these locations may be related to the nutritionally more diverse iron and magnesium-rich mafic substrates compared with the granites, and/or they may be sites of enhanced fluid flow at lithological interfaces.

### Different Communities Inhabit Different Impact Lithologies

The deep subsurface microbiome below the Chicxulub impact crater was dominated by Proteobacteria, Bacteroidota, Firmicutes, and Actinobacteria, similar to predominant microbial communities associated with deep subsurface igneous rocks such as basalt, granite, volcanic glass, and inactive hydrothermal vents (Jørgensen and Zhao, 2016; Zhang et al., 2016; Dutta et al., 2018; Hou et al., 2020). Despite a seemingly unequal downcore distribution of the ASVs, ordination and statistical analysis revealed that each lithology (postimpact, suevite, and granite) hosted a significantly different microbial community. The majority of ASVs in the 62 studied samples were not shared between the three lithologies with 23 taxa that were common to all lithologies, including taxa affiliated with Proteobacteria, Actinobacteria, and Bacteriodota. However, the relative abundance of some of the shared ASVs was different among the lithologies. Indicator species analysis revealed that some ASVs were only present in distinct core sections confirming that postimpact microbial niche separation has occurred in all three lithologies. The observed highest microbial diversity in addition to elevated cell numbers at the suevite to postimpact sedimentary transition further substantiates previous observations that an enhanced fluid flow and increased substrate availability at lithological boundaries and within high porosity deposits can provide long-term support for microbial growth (e.g., Parkes et al., 2005; Tanikawa et al., 2018).

### General Potential Microbial Metabolic Traits Occurring in the Oligotrophic Chicxulub Subsurface System

Although our research was based on 16S rRNA gene analyses, it may be possible to predict the main metabolic functions of key members of the microbial communities found in the different lithologies based on their taxonomic affiliations with closely related 16S rRNA gene sequences located on sequenced genomes from cultured relatives, environmental single-cell amplified genomes, and/or environmental shotgun metagenomes available from public databases. Our sequencing data revealed that the presence of archaea was very low (less than 0.5% of total reads), and no ASVs related to methanogenic archaea were found. ASVs related to putative dissimilatory sulfate-reducing Kapabacteria were detected in the lithologies, which may outcompete methanogenic archaea for substrates (Sela-Adler et al., 2017).

Most of the recovered ASVs were related to metabolically versatile bacteria involved in metal, nitrogen and sulfur cycling. For example, members of the genus *Paracoccus* (Alphaproteobacteria; Rhodobacteriales) are capable of using organic compounds, sulfur and iron as electron donors and nitrate as the electron acceptor (Kumaraswamy et al., 2006). Several ASVs from taxonomic groups that contain members of known denitrifiers were identified and belonged to Gammaproteobacteria (*Halomonas*, *Stenotrophomonas*, and *Methylorubrum*) as well as to Alphaproteobacteria (*Roseobacter*, *Roseomonas*, and Stappiaceae) (e.g., Weber and King, 2007; Yu et al., 2009; González-Domenench et al., 2010). Recently, it was suggested that sulfur-driven autotrophic denitrification could be the dominant process to sustain microbial survival in oligotrophic deep subsurface environments (Lau et al., 2016). Sulfur oxidizing bacteria (SOB) need to be present to drive this process, which would possibly be represented in Chicxulub’s deep subsurface rocks by *Pseudomonas* (Gammaproteobacteria), Rhodobacteriales, *Paracoccus* (Alphaproteobacteria), and Desulfobacteria (Desulfobacterota). Combined, our results suggest that a substantial part of the microbial community is involved in sulfur oxidation and denitrification under anaerobic conditions.

### Potential Metabolic Traits Associated With Different Impact Lithologies

SIMPER analyses showed that TOC and sulfur are the most important measured quantitative geochemical parameters that separate the postimpact sedimentary rocks from the two other lithologies, with TOC and sulfur being higher in the postimpact sedimentary rocks. Possibly ancient refractory organic matter is still available as carbon and energy sources for the residing microbial communities such as chemoheterotrophic Flavobacteria (e.g., *Tenacibaculum*), which were identified as significant indicator species (#160, 182) for this core section. Sulfur compounds may still be available to microbial communities, notably *Halomonas* spp. (indicator #641) since representatives of this genus are known to utilize nitrate to oxidize thiosulfate and sulfide under anaerobic condition in deep sea sediments (Teske et al., 2000). The detection of sequences affiliated with sulfate-reducing genera (Desulfobacterota) in the suevite and granitic rocks is consistent with the isolation of sulfate-reducing bacteria from both of these layers. The presence of sulfide minerals within the Chicxulub peak ring that carry an isotopic signature of microbial sulfate-reduction has been interpreted to be a signature of sulfate reduction in the hydrothermal system that formed immediately after impact (Kring et al., 2021). Our data suggest that sulfate reduction remains an active metabolism within the crater in the present day.

SIMPER analysis also shows the importance of porosity as a dominant factor in shaping the suevite communities and secondary to temperature as a factor shaping the communities in the granitic layer. This observation may be linked to the improvement of fluid flow and thus access to nutrients and energy in the higher porosity regions. As porosity is one of the key physical characteristics altered by impact, these data further corroborate the observation that the impact played a role in shaping the present-day community composition.

Microbial taxa significant for the high porosity suevite layers were closely related (99–100% sequence homology) to thermophilic microbes found in hot springs from Yellowstone Park, United States (Hugenholtz et al., 1998), Geyser valley, Kamchatka (Lee et al., 2012) and fumaroles from Hawaii (Wall et al., 2015). A thermophilic lifestyle of the indigenous microbial communities in the suevite is expected since *in situ* temperatures in this interval ranges between 50 and 55°C. This condition would be consistent with our enrichment of organisms at 50°C. The thermophilic members that were present in the crater lithologies at the time of sampling might stem from ancient hydrothermal systems that established in the crater and prevailed for more than 2 million years after impact (Kring et al., 2020, 2021). For example, one of the indicator species (#208) for the suevite interval belongs to Kapabacteria. This class was initially described as a novel clade (OPB56) related to non-photosynthetic Chlorobi from hot springs, which expressed genes involved in dissimilatory sulfate reduction (Thiel et al., 2019). *Meiothermus* (Deinococcus/Thermus clade) (Lee et al., 2012 and references therein) are also predominant significant indicator species for the suevite, albeit that these moderate thermophiles were also present at lower relative abundance in analyzed intervals from the postimpact interval. *Meiothermus* spp. are mostly known as aerobic heterotrophs but may also be indigenous members of the anaerobic deep subsurface biosphere as facultative anaerobes that can use alternate electron acceptors, notably nitrate (Rempfert et al., 2017).

Comamonadaceae was the most prominent indicator family (#63) in the shattered granitic basement and its pre-impact, subvolcanic, mafic dikes. Members of this family have shown to be able to oxidize hydrogen and have been reported in hydrogen-enriched subsurfaces and ophiolitic rock sequences (Nyyssönen et al., 2014; Purkamo et al., 2015). Furthermore, Xanthomonadales were specific indicators for the granitic basement. Members of this order have been associated with sedimentary dark CO_2_ fixation while being involved in sulfide oxidation (Dyksma et al., 2016). More specifically, *Luteimonas* (indicator taxon #694) strains, previously isolated from the subseafloor of the South Pacific Gyre, have the genomic potential to be capable of thriving under extreme conditions including hypersaline and toxic environments (Zhang et al., 2015). Although in low abundance, Bathyarchaeota were exclusively detected in the granitic basement. These hyperthermophilic anaerobic archaea are abundant in anoxic sediments and are potentially involved in the degradation of aromatic compounds (Zhou et al., 2018).

The data we acquired from the granitic rocks are similar to results collated from basaltic oceanic crust buried beneath sediments (Jørgensen and Zhao, 2016) where cell numbers were ∼10^4^/g of rock and the microbial communities were dominated by Proteobacteria, in particular Alpha and Gammaproteobacteria, Actinobacteria, and Bacteriodetes. These data suggest that these taxa constitute dominant components of both oligotrophic basaltic and granitic subsurface environments.

### Deep Biosphere and Planetary Science Significance

The data on the microbiology of the present-day Chicxulub impact crater have allowed us to make the first observations of how the impactor that caused the end-Cretaceous mass extinction led to a modern deep biosphere. The microbial abundances vary with the crater arrangements of lithological and structural units and show enhancements at impact-induced interfaces. The crater units also harbor distinct microbial communities with diverse metabolic capabilities.

Surface-dwelling biota, particularly microbial communities, recover rapidly from catastrophic changes. At the site of the Chicxulub impact, a high productivity marine ecosystem was established within 30 kyr (Lowery et al., 2018; Bralower et al., 2020; Schaefer et al., 2020). Although we do not know when the present-day deep biosphere at Chicxulub became established since biotic turnover likely occurred since the impact, both the abundance and diversity of the deep biosphere in the Chicxulub crater is still structured by the geological changes wrought in the first hours and days of the Cenozoic (Gulick et al., 2019). This includes habitat enhancement in suevites and in impact-induced geological interfaces in the otherwise biologically impoverished granitic rocks.

Finally, we note that our observations have application to the search for life on other planetary bodies, particularly Mars. The deep subsurface at Chicxulub shows that large impacts generate deep geological interfaces, favoring fluid flow, enhancing mineral diversity and thus the accessibility of nutrients and energy in the subsurface. A substantial number of large impact craters on Mars have been preserved since its early history because of the lack of plate tectonics (Michalski and Niles, 2010; Robbins and Hynek, 2012; Squyres et al., 2012). Although in contrast to Chicxulub, the Martian crust is basaltic, in analogy, the fractured, porous subsurface of large impact craters on Mars are propitious places to focus scientific exploration missions to investigate habitable environments and to test the hypothesis of the presence of life on Mars.

## Data Availability Statement

The datasets presented in this study can be found in online repositories. The names of the repository/repositories and accession number(s) can be found below: NCBI BioProject, accession no: PRJNA726950.

## Author Contributions

MJLC, CC, AW, and LS collected and processed the offshore samples with participation of all science party members during IODP-ICDP Expedition 364. CC, BS, CW, MJLC, and KG wrote the manuscript with contributions from all other authors. CC performed cell counts and incubation the experiments. BS carried out the experiments. BS and CW carried out statistical analysis of the microbial community analysis. JL provided down-hole porosity data and petrographic analyses were performed by AW. All authors contributed to the article and approved the submitted version.

## Conflict of Interest

The authors declare that the research was conducted in the absence of any commercial or financial relationships that could be construed as a potential conflict of interest.

## Footnotes

1 http://www.qiime2.org/

2 http://genevenn.sourceforge.net

## Supplementary Material

Supplementary Figure 1 Downcore distributions of **(A)** S, **(B)** Fe, **(C)** Mn (wt%), and **(D)** temperature (°C).

Supplementary Figure 2 Cell enumerations in drilling mud mix.

Supplementary Figure 3 Canonical analysis of principal coordinates (CAP) showing the spatial distribution of all 2,757 ASVs that were recovered from the core samples as well as from the various controls for contamination.

Supplementary Figure 4 Bar graph showing the relative abundance of the major phyla in samples from the three lithologies and in the corresponding drilling muds.

Supplementary Figure 5 Abundances of microbial taxa.

Supplementary Figure 6 Relative abundance of ASVs at phylum (% of total phyla) and class (% of the corresponding phylum) levels.

Supplementary Table 1 Lithology associated with the sampled core depths.

Supplementary Table 2 ASV sequences shown in the major lithological intervals in the Chicxulub crater.

Supplementary Table 3 Pairwise permutational multivariate analysis of variance (PERMANOVA) for the relationship between microbial community composition (Bray-Curtis similarity of standardized and square root transformed) and lithology type.

Supplementary Table 4 Identity of significant indicator species in the major lithological intervals in the Chicxulub crater.

Supplementary Table 5 Identity of significant indicator species in the major lithological intervals in the Chicxulub crater resulting from pairwise indicator species analysis.

Supplementary Table 6 SIMPER analysis of contributions of major environmental parameters to microbial community composition.
