# Supplementary material for: Shaping of the Present-Day Deep Biosphere at Chicxulub by the Impact Catastrophe That Ended the Cretaceous
Source: Front Microbiol. 2021 Jun 24;12:668240. doi: 10.3389/fmicb.2021.668240 (PMC8264514; doi:10.3389/fmicb.2021.668240)

**Supplementary Materials for**

**Shaping of the present-day deep biosphere at Chicxulub by the impact catastrophe that ended the Cretaceous**

Charles S. Cockell* Bettina Schaefer, Cornelia Wuchter, Marco J. L. Coolen*, Kliti Grice, Luzie Schnieders, Joanna V. Morgan, Sean P.S. Gulick, Axel Wittmann, Johanna Lofi, Gail Christeson, David A. Kring, Michael Whalen, Timothy Bralower, Gordon Osinski, Philippe Claeys, Pim Kaskes, Sietze de Graaff, Thomas Dehais, Steven Goderis, Natali Hernandez Becerra, Sophie Nixon and IODP-ICDP Expedition 364 Scientists.

*corresponding author: c.s.cockell@ed.ac.uk or marco.coolen@curtin.edu.au

**This PDF includes:**

**Figure 1**. Downcore distributions of (a) S, (b) Fe, (c) Mn (wt%), and (d) temperature (°C).

**Figure 2.** Cell enumerations in drilling mud mix.

**Figure 3.** Canonical analysis of principal coordinates (CAP) showing the spatial distribution of all 2757 ASVs that were recovered from the core samples as well as from the various controls for contamination.

**Figure 4.** Bar graph showing the relative abundance of the major phyla in samples from the three lithologies and in the corresponding drilling muds.

**Figure 5.** Abundances of microbial taxa.

**Figure 6.** Relative abundance of ASVs at phylum (% of total phyla) and class (% of the corresponding phylum) levels.

**Table 1.** Lithology associated with the sampled core depths.

**Table 2.** ASV sequences shown in the major lithological intervals in the Chicxulub crater.

**Table 3.** Pairwise permutational multivariate analysis of variance (PERMANOVA) for the relationship between microbial community composition (Bray-Curtis similarity of standardized and square root transformed) and lithology type.

**Table 4.** Identity of significant indicator species in the major lithological intervals in the Chicxulub crater.

**Table 5.** Identity of significant indicator species in the major lithological intervals in the Chicxulub crater resulting from pairwise indicator species analysis.

**Table 6.** SIMPER analysis of contributions of major environmental parameters to microbial community composition.

**
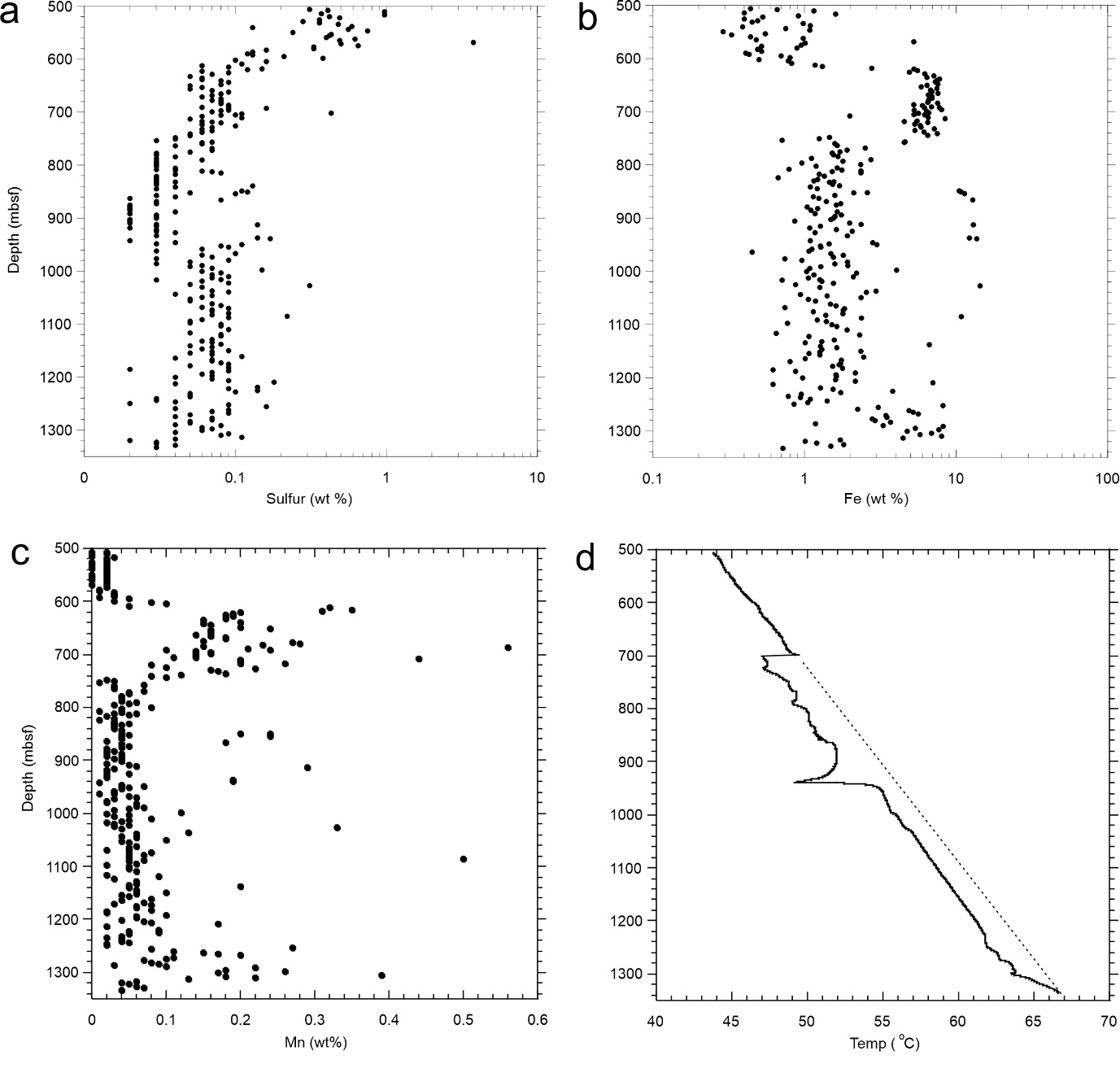
**

**Supplementary Figure 1.** Downcore distribution of (a) S, (b) Fe, (c) Mn (wt%), and (d) borehole fluid temperature (°C). The dashed line shows extrapolated borehole temperature (see Methods for detail).

**
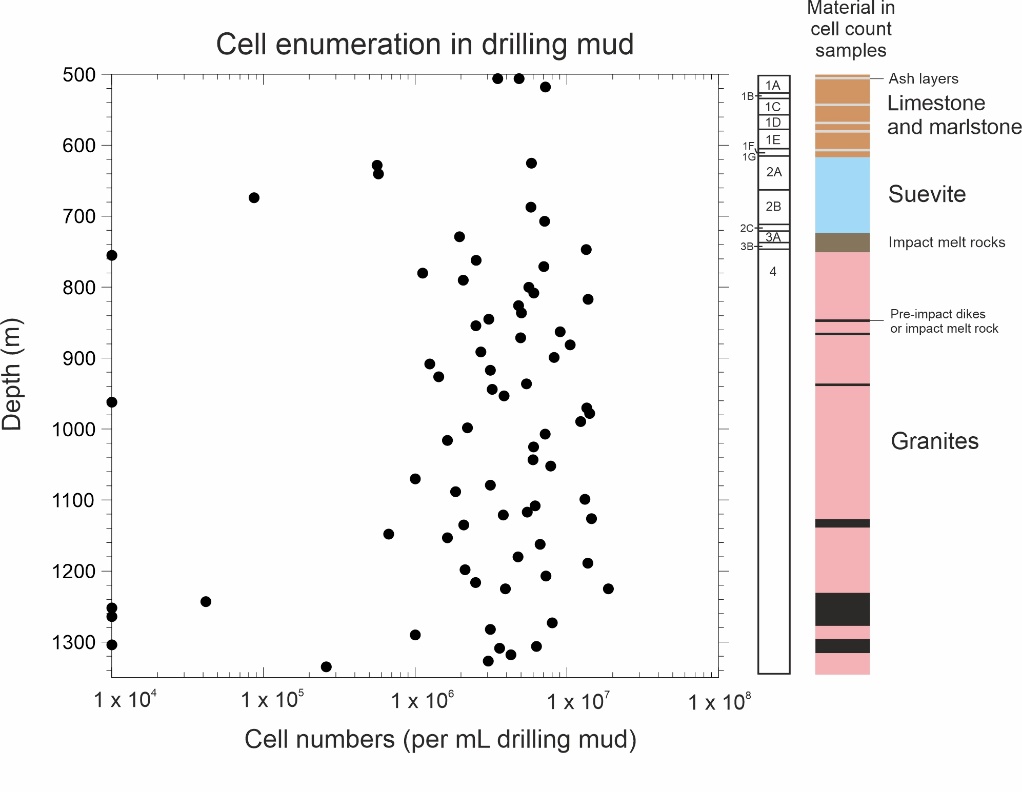
**

**Supplementary Figure 2.** **Microbial enumerations in drilling mud.** Drilling mud cell numbers (per mL of drilling mud shown with respect to depth).

**
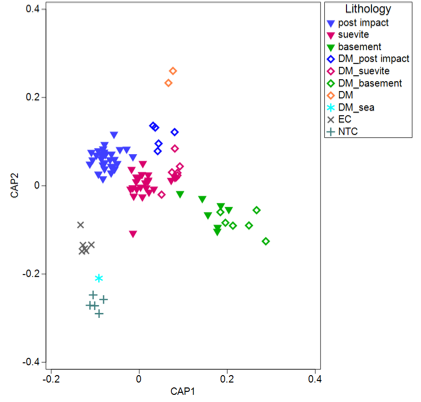
**

**Supplementary Figure 3.** Canonical analysis of principal coordinates (CAP) showing the spatial distribution of all 2757 ASVs that were recovered from the core samples as well as from the various controls for contamination: drilling mud (DM), seawater, extraction controls (EC), and non-template controls (NTC). This analysis was performed on standardized and square root transformed data using Bray-Curtis resemblance matrix.


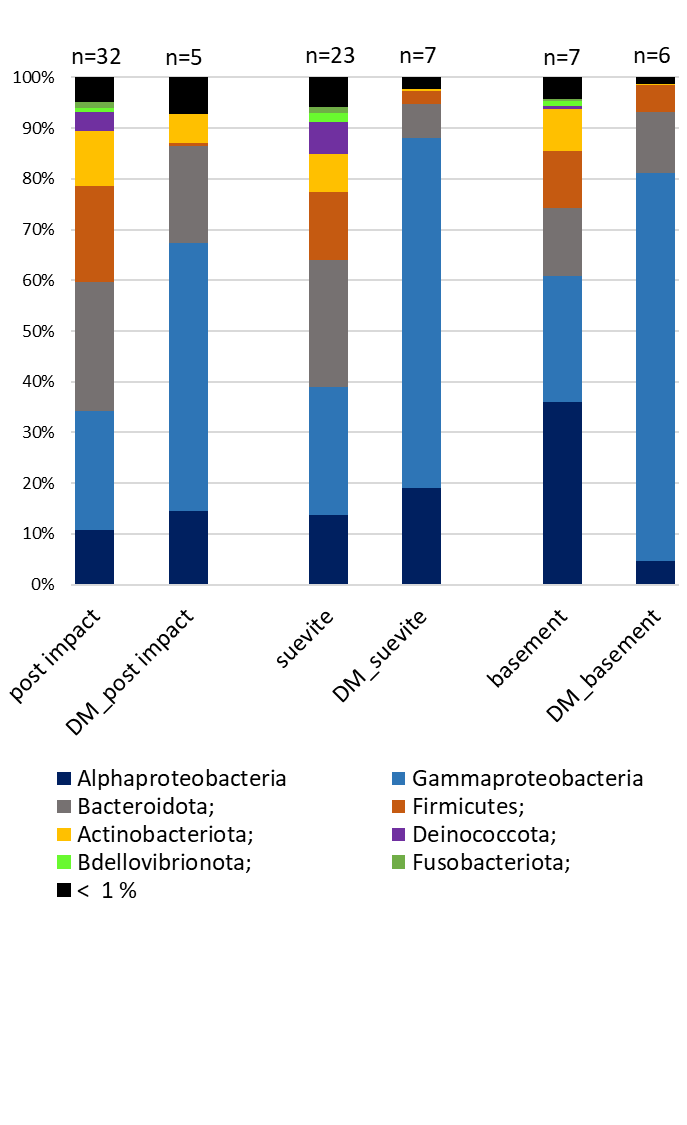


**Supplementary Figure 4.** Bar graph showing the relative abundance of the major phyla in samples from the three lithologies and in the corresponding drilling muds. The Proteobacterial classes Alpha- and Gammaproteobacteria are shown separately.

**
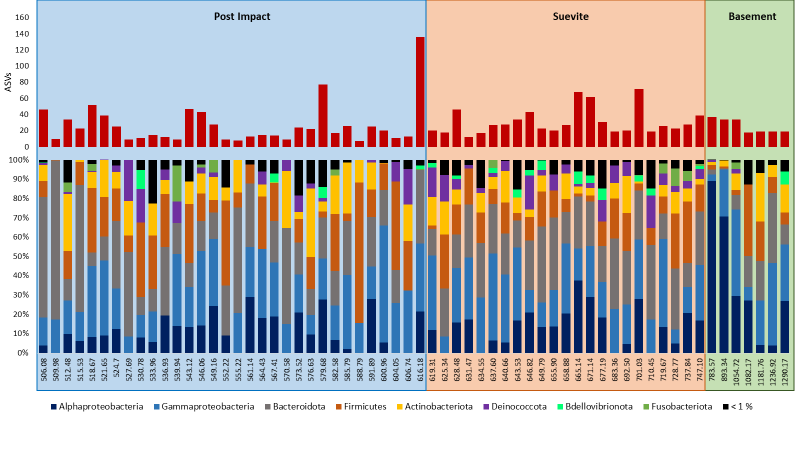
**

**Supplementary Figure 5.** Abundances of microbial taxa. (A) Bar graph showing the number of taxa per sample. Here, a taxon is the sum of ASVs that were assigned to the same lowest taxonomic level. The highest number of taxa was found at the transition from suevite to post impact Cenozoic marine sediment at 616.8 mbsf. (B) Relative abundance of the major phyla. The relative abundance of Proteobacteria is shown at class level (Alpha- and Gammaproteobacteria).

**
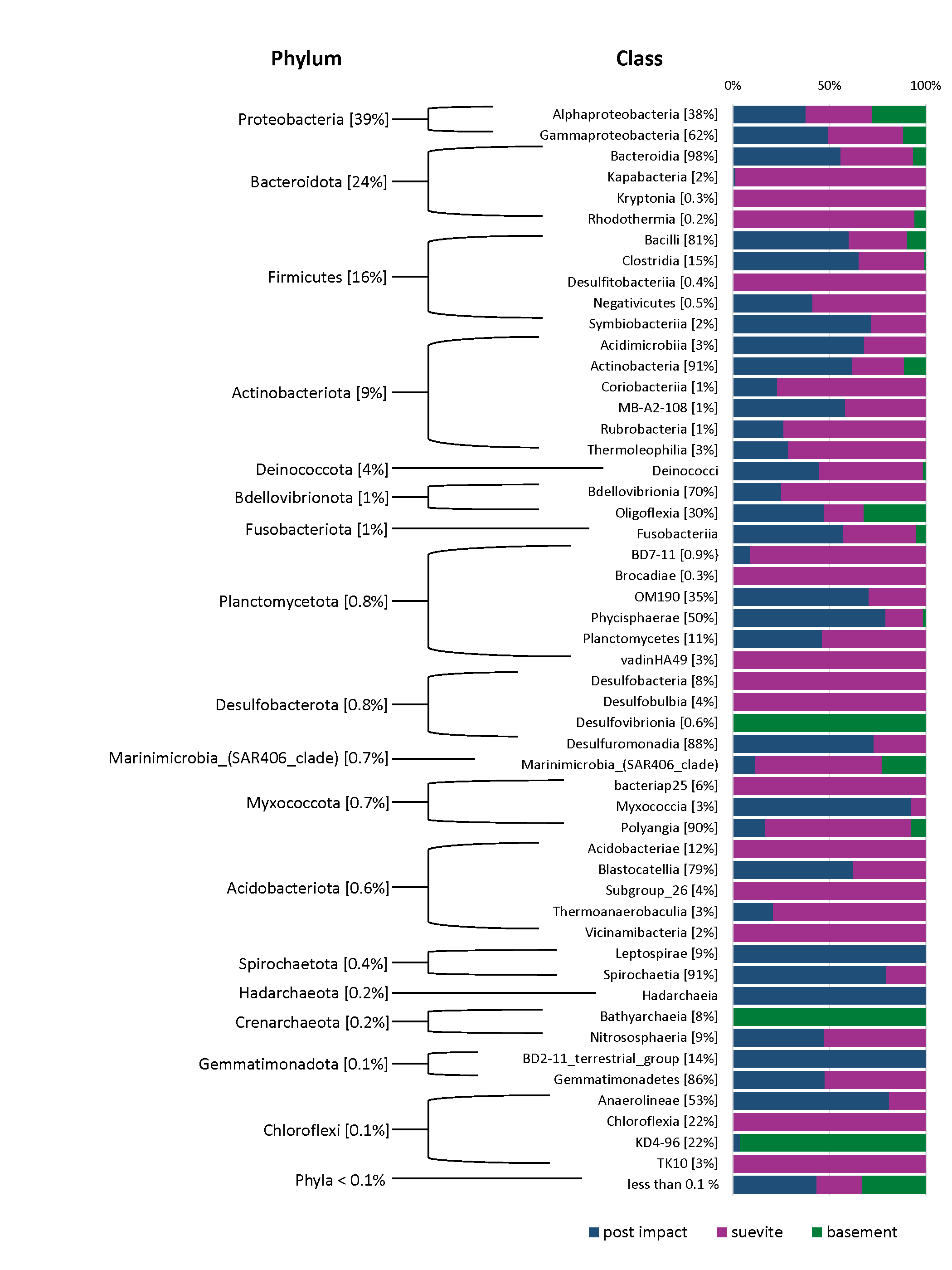
**

**Supplementary Figure 6.** Relative abundance of ASVs at phylum (% of total phyla) and class (% of the corresponding phylum) levels. Shown are averages percentages for all samples combined. The bar graph indicates the average relative abundance of each class in the different lithologies.

**Supplementary Table 1.** Lithology associated with the sampled core depths.

| **Core** | **Sample depth (top**  **of sample) [mbsf]** | **Major stratigraphic unit** | **Lithologic Description** |
| --- | --- | --- | --- |
| 2 | 506.08 | Post-impact sed. rock | Laminated medium brown limestone |
| 4 | 509.98 | Post-impact sed. rock | Sandy, yellow-grey sediment or laminated, dark-brown limestone |
| 7 | 518.67 | Post-impact sed. rock | Laminated, medium-brown limestone |
| 10 | 527.69 | Post-impact sed. rock | Laminated, light-brown limestone |
| 13 | 536.93 | Post-impact sed. rock | Hummocky, medium-brown limestone |
| 15 | 542.81 | Post-impact sed. rock | Hummocky, medium-brown limestone |
| 16 | 546.06 | Post-impact sed. rock | Laminated, dark-brown limestone |
| 18 | 552.22 | Post-impact sed. rock | Laminated, dark-brown limestone |
| 22 | 564.43 | Post-impact sed. rock | Laminated, dark-brown limestone |
| 25 | 573.52 | Post-impact sed. rock | Hummocky, medium-brown limestone |
| 26 | 576.13 | Post-impact sed. rock | Cross-bedded medium-brown limestone |
| 27 | 579.68 | Post-impact sed. rock | Convoluted yellow limestone |
| 28 | 582.58 | Post-impact sed. rock | Convoluted (possibly bioturbated) yellow-grey limestone |
| 31 | 591.89 | Post-impact sed. rock | White, bioturbated limestone |
| 34 | 600.96 | Post-impact sed. rock | White nodular limestone |
| 39 | 616.18 | Post-impact sed. rock | White stylotilized limestone |
| 40 | 619.31 | Suevite | Very fine-grained, sorted suevite |
| 42 | 625.34 | Suevite | Fine-grained, sorted suevite |
| 43 | 628.48 | Suevite | Very fine-grained, sorted suevite |
| 44 | 631.47 | Suevite | Fine-grained, sorted suevite |
| 45 | 634.55 | Suevite | Fine-grained, sorted suevite |
| 46 | 637.6 | Suevite | Very fine-grained, sorted suevite |
| 47 | 640.66 | Suevite | Fine-grained, sorted suevite |
| 48 | 643.53 | Suevite | Medium-fine-grained, sorted suevite |
| 49 | 646.82 | Suevite | Medium-grained, sorted suevite |
| 50 | 649.79 | Suevite | Medium-grained, sorted suevite |
| 52 | 655.9 | Suevite | Medium-grained, sorted suevite |
| 53 | 658.88 | Suevite | Medium-fine-grained, sorted suevite below red alteration veins |
| 55 | 665.14 | Suevite | Medium-fine-grained, sorted suevite |
| 57 | 671.14 | Suevite | Medium-grained, sorted suevite |
| 59 | 677.19 | Suevite | Medium-coarse grained, sorted suevite |
| 62 | 683.36 | Suevite | Medium-coarse grained, sorted suevite |
| 68 | 692.5 | Suevite | Coarse grained, sorted suevite |
| 73 | 701.03 | Suevite | Coarse grained, sorted suevite |
| 81 | 710.45 | Suevite | Impact melt body in coarse grained, sorted suevite |
| 86 | 719.67 | Suevite | Unsorted suevite with green groundmass |
| 89 | 728.77 | Suevite | Dark impact melt rock with green schlieren and brecciation zones |
| 92 | 737.84 | Suevite | Dark, clast-bearing impact melt rock |
| 95 | 747.1 | Granitic basement | Cataclased granite with grey breccia veins, 5 cm below contact to dark impact melt rock. |
| 98 | 756.12 | Granitic basement | Dark, clast-bearing impact melt rock |

| **Core** | **Sample depth (top**  **of sanple) [mbsf]** | **Major stratigraphic unit** | **Lithologic Description** |
| --- | --- | --- | --- |
| 102 | 765.29 | Granitic basement | Faulted granite |
| 105 | 774.41 | Granitic basement | Faulted granite |
| 108 | 783.57 | Granitic basement | Pegmatitic, faulted granite |
| 111 | 792.8 | Granitic basement | Granite with steeply dipping grey veins (cataclasite/altered pseudotchylite?) |
| 114 | 801.87 | Granitic basement | Faulted granite |
| 118 | 811.04 | Granitic basement | Granite |
| 122 | 820.24 | Granitic basement | Granite |
| 125 | 829.29 | Granitic basement | Faulted granite |
| 131 | 838.2 | Granitic basement | Faulted granite |
| 134 | 847.57 | Granitic basement | Basanite/dolerite dike with granite inclusion. |
| 140 | 856.4 | Granitic basement | Faulted granite |
| 143 | 865.2 | Granitic basement | Basanite/dolerite dike |
| 146 | 874.53 | Granitic basement | Faulted granite |
| 149 | 883.86 | Granitic basement | Granite |
| 153 | 893.34 | Granitic basement | Granite |
| 156 | 902.46 | Granitic basement | Faulted granite |
| 159 | 902.57 | Granitic basement | Faulted granite |
| 164 | 920.82 | Granitic basement | Trachyte subvolcanic dike |
| 167 | 929.95 | Granitic basement | Faulted granite |
| 170 | 938.29 | Granitic basement | Basanite/dolerite dike |
| 173 | 947.79 | Granitic basement | Faulted granite |
| 176 | 954.36 | Granitic basement | Intensely faulted granite |
| 180 | 963.52 | Granitic basement | Granite |
| 183 | 972.79 | Granitic basement | Intensely shear-faulted, partly cataclased granite |
| 186 | 981.89 | Granitic basement | Faulted granite |
| 189 | 990.98 | Granitic basement | Granite |
| 192 | 1000.18 | Granitic basement | Intensely faulted, partly brecciated granite |
| 194 | 1005.85 | Granitic basement | Granite-grey / clastic dike |
| 199 | 1018.45 | Granitic basement | Crenulation-faulted granite |
| 202 | 1026.69 | Granitic basement | Intensely sheared and brecciated granite with dark melt veins |
| 205 | 1036 | Granitic basement | Faulted granite with dark/greenish alteration typical for alteration zones surrounding pre-impact dikes |
| 208 | 1045.46 | Granitic basement | Faulted granite |
| 211 | 1054.72 | Granitic basement | Faulted granite with dark/greenish alteration typical for alteration zones surrounding pre-impact dikes |
| 214 | 1063.6 | Granitic basement | Intensely deformed, "greened" granite near contact to cataclasite-  zone that contains dark melt |
| 217 | 1073.01 | Granitic basement | Faulted granite |
| 220 | 1082.17 | Granitic basement | Granite |
| 223 | 1090.68 | Granitic basement | Shear-faulted granite, sample contains fault-gouge |
| 226 | 1100.06 | Granitic basement | Faulted granite with dark/greenish alteration typical for alteration zones surrounding pre-impact dikes |
| 229 | 1109.36 | Granitic basement | Faulted granite |
| 232 | 1118.6 | Granitic basement | Crenulation-faulted, partly cataclased granite |
| 235 | 1124.79 | Granitic basement | Shatter-coned phonotephrite dike |
| 238 | 1137.02 | Granitic basement | Shatter-coned phonotephrite dike |
| 241 | 1146.39 | Granitic basement | Faulted granite/aplite |

| **Core** | **Sample depth (top**  **of sanple) [mbsf]** | **Major stratigraphic**  **unit** | **Lithologic Description** |
| --- | --- | --- | --- |
| 244 | 1154.52 | Granitic basement | Granite |
| 247 | 1163.02 | Granitic basement | Granite/aplite |
| 250 | 1172.43 | Granitic basement | Faulted granite |
| 253 | 1181.76 | Granitic basement | Granite |
| 256 | 1191 | Granitic basement | Faulted, pegmatitic granite |
| 259 | 1200.23 | Granitic basement | Granite |
| 262 | 1209.05 | Granitic basement | Faulted, amethyst-bearing granite/aplite |
| 265 | 1218.4 | Granitic basement | Intensely crenulation-faulted, dark-melt-veined granite |
| 268 | 1227.6 | Granitic basement | Cataclased and crenulation-faulted granite |
| 271 | 1236.92 | Granitic basement | Intensely faulted, amethyst-bearing granite |
| 274 | 1246.19 | Granitic basement | Crenulation-faulted and cataclased granite |
| 277 | 1255.57 | Granitic basement | Suevite |
| 280 | 1264.86 | Granitic basement | Suevite |
| 283 | 1274.2 | Granitic basement | Suevite |
| 286 | 1283.48 | Granitic basement | Amethyst-bearing granite clast in suevite-veined granite block |
| 289 | 1291.17 | Granitic basement | Dark impact melt rock with green schlieren and brecciation zones |
| 292 | 1300.64 | Granitic basement | Dark mylonite clast in clast-rich impact melt rock/melt-rich suevite |
| 295 | 1309.86 | Granitic basement | Clast-rich impact melt rock |
| 298 | 1319.21 | Granitic basement | Faulted, feldspar-rich granite |
| 301 | 1328.51 | Granitic basement | Crenulation-faulted, feldspar-rich granite |
| 303 | 1332.97 | Granitic basement | Sheared and crenulation-faulted, feldspar-rich granite |

**Supplementary Table 3.** Pairwise permutational multivariate analysis of variance (PERMANOVA) for the relationship between microbial community composition (Bray-Curtis similarity of standardized and square root transformed) and lithology type. The analysis was run using the Monte-Carlo permutation procedure with 999 permutations in PRIMER E vs. 7.


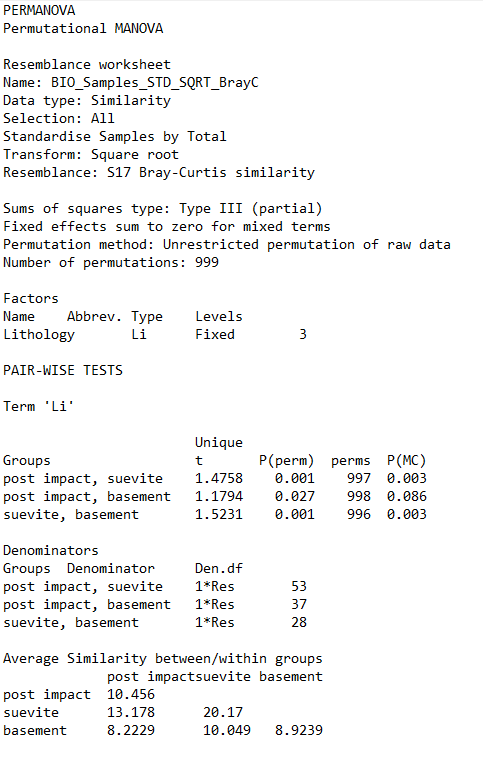


**Supplementary Table 4.** Identity of significant indicator species in the major lithological intervals in the Chicxulub crater. Indicator taxa are shown at genus level or the next taxonomic level at which identification was possible. Note that ISA #160,182, and 641 were obtained from pairwise ISA (Table S3)


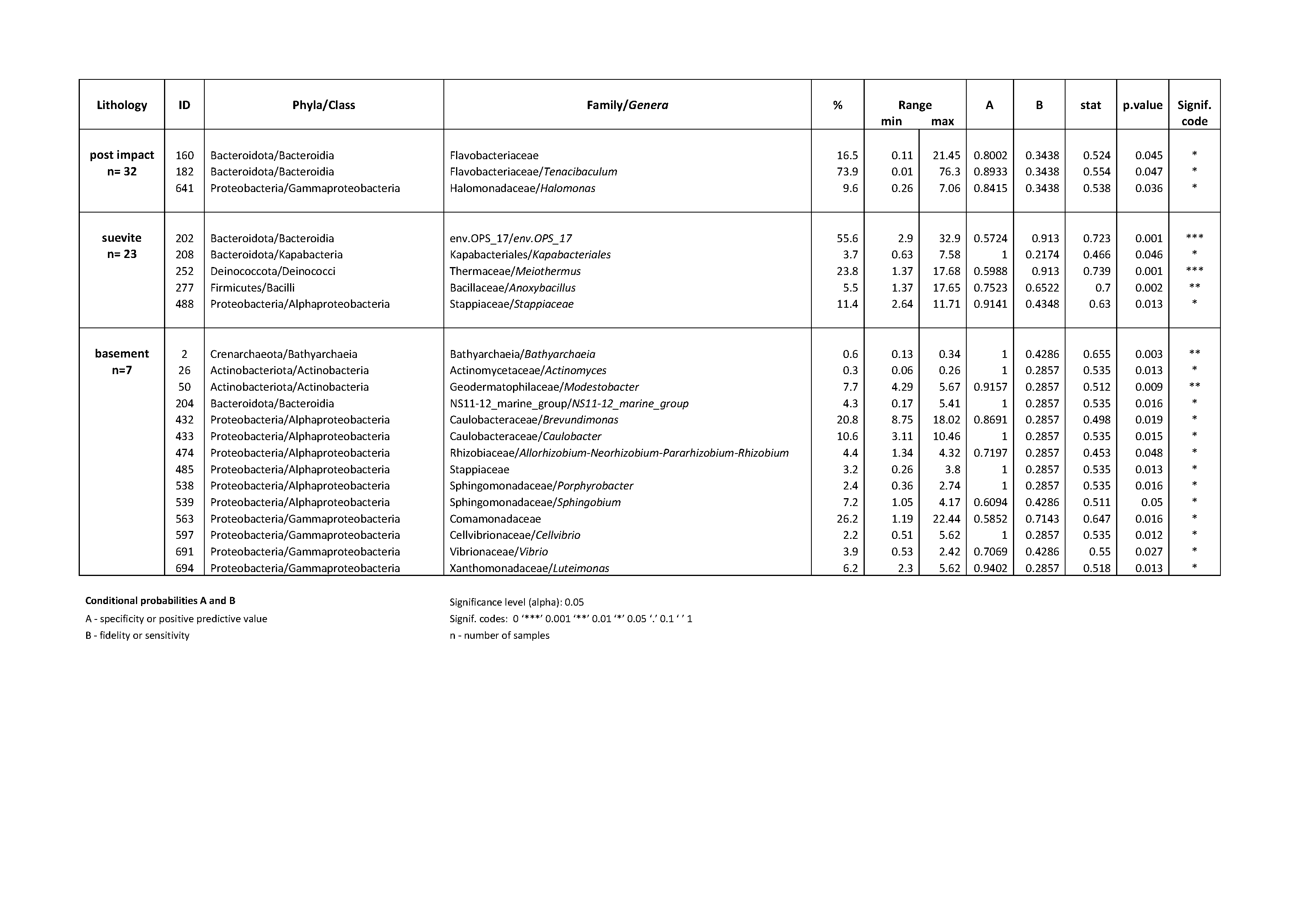


**Supplementary Table 5.** Identity of significant indicator species in the major lithological intervals in the Chicxulub crater resulting from pairwise indicator species analysis. Indicator taxa are shown at genus level or the next taxonomic level at which identification was possible.


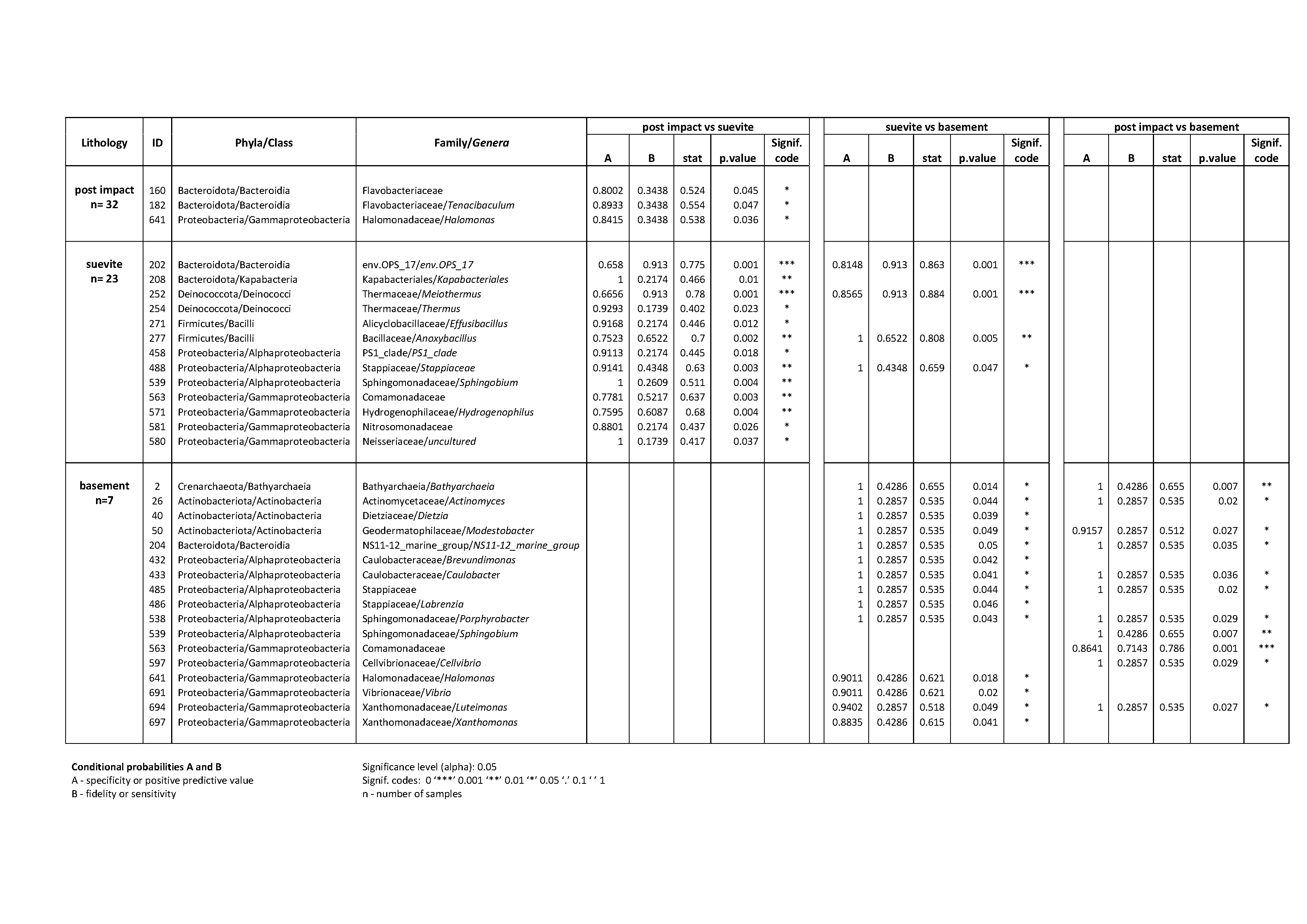


**Supplementary Table 6.** SIMPER analysis of contributions of major environmental parameters to microbial community composition. Analysis is shown for the three major crater lithologies.


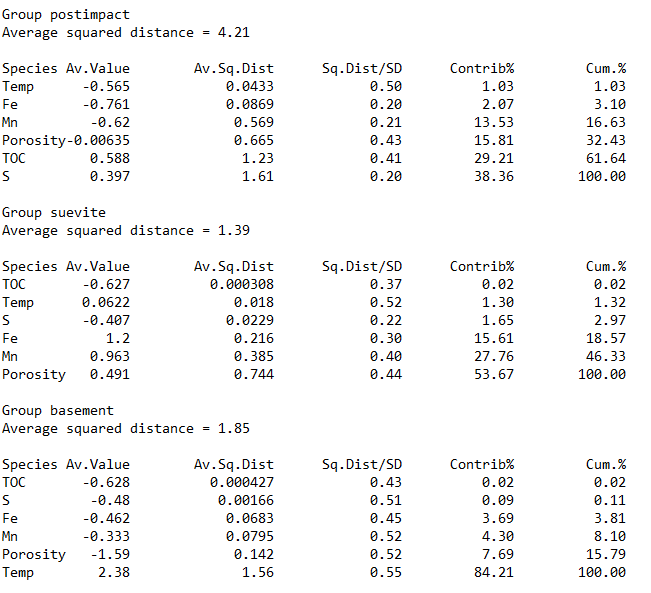

Supplement: Supplementary Figure 1 — Downcore distributions of (A) S, (B) Fe, (C) Mn (wt%), and (D) temperature (°C). [file Data_Sheet_1.DOCX]
